# Primary care visits due to injuries among the Aboriginal off-reserve population of British Columbia, Canada, 1991–2010

**DOI:** 10.1186/s12939-015-0269-5

**Published:** 2015-11-19

**Authors:** Andrew Jin, M. Anne George, Mariana Brussoni, Christopher E. Lalonde, Rod McCormick

**Affiliations:** Epidemiology consultant, Surrey, British Columbia Canada; Department of Pediatrics, Faculty of Medicine, University of British Columbia, Vancouver, British Columbia Canada; Child and Family Research Institute, Vancouver, British Columbia Canada; School of Population and Public Health, University of British Columbia, Vancouver, British Columbia Canada; Department of Psychology, Faculty of Social Sciences, University of Victoria, Victoria, British Columbia Canada; Faculty of Human, Social and Educational Development, Thompson Rivers University, Kamloops, British Columbia Canada; Current address: Child and Family Research Institute, Room F508, 4480 Oak Street, Vancouver, BC V6H 3V4 Canada; Current address: BC Injury Research & Prevention Unit, Vancouver, British Columbia Canada

**Keywords:** Wounds and injuries (MeSH), Indians, North American (MeSH), Indigenous population (MeSH), “First Nations”, British Columbia (MeSH), Canada (MeSH), Epidemiology (MeSH), Population surveillance (MeSH)

## Abstract

**Background:**

Aboriginal people in British Columbia (BC) have higher injury incidence than the general population. This report describes variability in visits to primary care due to injury, among injury categories, time periods, geographies, and demographic groups.

**Methods:**

We used BC’s universal health care insurance plan as a population registry, linked to practitioner payment and vital statistics databases. We identified Aboriginal people by insurance premium group and birth and death record notations. Within that population we identified those residing off-reserve according to postal code. We calculated crude incidence and Standardized Relative Risk (SRR) of primary care visit due to injury, standardized for age, gender and Health Service Delivery Area (HSDA), relative to the total population of BC.

**Results:**

During 1991 through 2010, the crude rate of primary care visit due to injury in BC was 3172 per 10,000 person-years. The Aboriginal off-reserve rate was 4291 per 10,000 and SRR was 1.41 (95 % confidence interval: 1.41 to 1.42). Northern and non-metropolitan HSDAs had higher SRRs, within both total BC and Aboriginal off-reserve populations. In every age and gender category, the HSDA-standardized SRR was higher among the Aboriginal off-reserve than among the total population. For all injuries combined, and for the categories of trauma, poisoning, and burn, between 1991 and 2010, crude rates and SRRs declined substantially, but proportionally more rapidly among the Aboriginal off-reserve population, so the gap between the Aboriginal off-reserve and total populations is narrowing, particularly among metropolitan residents.

**Conclusions:**

These findings corroborate our previous reports regarding hospitalizations due to injury, suggesting that our observations reflect real disparities and changes in the underlying incidence of injury, and are not merely artefacts related to health care utilization.

## Background

Aboriginal people in British Columbia (BC) have higher incidences of injuries than the general population [1–6]. Our project, *Injury in British Columbia’s Aboriginal Communities: Building Capacity while Developing Knowledge* [7–11], adds information about variability of incidence rates among injury categories, geographic regions, time periods, and demographic and socio-economic groups within the Aboriginal population. This report focuses on visits to primary care due to injuries.

Most previous researchers in Canada have studied injuries among the populations of Indian reserves [1, 5, 12, 13] or areas with high proportions of Aboriginal residents [14, 15], and exclude the experience of Aboriginal people who live elsewhere. In BC, such exclusion would be materially significant because 74 % of people who self-identified as Aboriginal in the 2006 Census did not reside in any recognized Indian reserve or settlement [16]. Aboriginal people may choose to live off-reserve for many reasons, including employment opportunities, pursuit of education, improved availability and quality of housing, need for specialized health care, and for some women, the need to escape domestic violence [17]. Plausibly, these factors could also be associated with differences in injury incidence and other health outcomes. A Canadian study of Potential Years of Life Lost (PYLL), among a cohort who self-identified as Aboriginal in the 1991 Census, found the rate of PYLL due to injury was 2.74 times higher among people with Indian status living off-reserve, and 4.09 times higher among those living on-reserve, compared to the rate among non-Aboriginal Canadians [18].

Two BC studies of mortality incidence used the province’s universal health care insurance program as a population registry, and identified Aboriginal people, within the population, and among death records, by record linkage, using a combination of insurance premium group, Indian status, and birth and death record notations [2, 4]. The method included both on-reserve and off-reserve residents, but the analyses did not separate or compare the mortality experience of the two groups. Also, these studies did not address how much of the differences in injury rates between the Aboriginal and general populations were due to the higher proportions of the Aboriginal population residing in northern, rural or remote locations that may be more conducive to injuries for a variety of reasons, i.e., harsher physical environment, more hazardous occupations, less adherence to safety measures, hazardous outdoor recreational activities, transportation over longer distances and at higher speeds, or less access to emergency medical care [19].

We adapted these studies’ methods [2, 4], and made three specific improvements. First, we studied non-fatal injury-related events, which occur much more frequently than deaths due to injury, thus enabling more precise description of variability in incidence rates among geographic regions, demographic groups and time periods. Second, we standardized comparisons of injury rates between the Aboriginal and general populations, by age, gender and also region of the province, thus compensating for the effects of northern location and degree of urbanization. Third, we distinguished between the on-reserve and off-reserve Aboriginal populations, and compared their injury experiences separately to that of the general population of BC.

Studying hospitalizations due to injury, we previously reported that during the period 1991 to 2010, the Standardized Relative Risk (SRR) of hospitalization due to unintentional falls was 1.77 among the Aboriginal off-reserve population and 2.00 among the Aboriginal on-reserve population [11]. The present analysis applies our improved method to the study of visits to primary care due to injury, among the off-reserve Aboriginal population of BC. Such a study has not previously been reported in the literature.

## Methods

### Ethics review and permission for data access

The University of British Columbia Behavioural Research Ethics Board reviewed and approved our methods (BREB file H06-80585). The Data Stewards representing the BC Ministry of Health and the BC Vital Statistics Agency approved the data access requests. We used existing databases, permanently linked by British Columbia Personal Health Number, maintained by Population Data BC (project George 11–012) [20–23]. Population Data BC rendered the client records anonymous before our analysis. **Disclaimer**: all inferences, opinions, and conclusions drawn in this journal manuscript are those of the authors, and do not reflect the opinions or policies of the Data Stewards.

### Population counts

We used the registration and premium billing files [20] of the Medical Services Plan of BC (MSP, the province’s universal health care insurance program), to count the total resident population of BC at the mid-points of fiscal years 1991–1992 through 2009–2010. Within this population, we identified people whom we considered “Aboriginal”, using a combination of insurance premium group, and notations of Indian status on linked birth [21] and death records [22]. We previously described this method, and discussed the quality of the population registry, and validity and limitations of the Aboriginal identification [10, 11].

We classified as “on-reserve” those Aboriginal people residing in a postal code area associated with an Indian reserve or settlement recognized by Statistics Canada and the federal Department of Aboriginal Affairs and Northern Development. We classified all other Aboriginal people as “off-reserve”.

There are sixteen Health Service Delivery Areas (HSDAs) in BC [24]. We classified HSDAs as “metropolitan” (HSDAs 22, 23, 31, 32, 33 and 41, comprising metropolitan Vancouver and metropolitan Victoria) or “not metropolitan” (HSDAs (11, 12, 13, 14, 21, 42, 43, 51, 52, and 53). Vancouver and Victoria are the two largest Census Metropolitan Areas in BC, containing 60.4 % of the population enumerated in BC by the 2011 Census of Canada [25]. The categories of “metropolitan” and “not metropolitan” are respectively the same as the categories we called “urban” and “not urban” in our previous reports [9–11].

We tabulated population counts by fiscal year, gender, 5-year age group, Aboriginal status, reserve residence, HSDA, and metropolitan residence.

### Primary care visit counts

We tabulated counts of visits to primary care [23] by residents of BC, from January 1, 1991 through December 31, 2010. We defined a “primary care visit as due to injury” as a *payment by MSP* for examination by a general practice physician, emergency physician, nurse practitioner, pediatrician, geriatrician, dentist or optometrist, with a diagnostic code indicative of injury, i.e., an International Classification of Diseases - Revision 9 (ICD-9) numeric code in the range 800 through 999, or an MSP code indicative of injury (listed in the Appendix). This definition excludes payments to surgeons, anaesthesiologists, radiologists and physical therapists because we consider these to be providers of diagnostic and secondary treatment procedures. Not included are payments to practitioners by Work Safe BC (BC’s mandatory occupational injury insurance and compensation program). Our definition may under-count examinations by emergency physicians and nurse practitioners, because these practitioners are often paid salaries or sessional (hourly) fees, but the under-counting would apply both to Aboriginal people residing off-reserve and to the general population, so there would be no bias in the comparison between these two populations (assuming that off-reserve Aboriginal and non-Aboriginal people access such practitioners equally). Our definition does, however, seriously under-count examinations by physicians and nurse practitioners employed by community health facilities located on Indian reserves, again because these practitioners are often paid salaries or sessional fees, and this under-counting would apply to people resident on-reserve, but not to those resident off-reserve (assuming that off-reserve Aboriginal people would not travel back to the reserve to obtain primary care). For this reason, the present analysis makes no comparison of Aboriginal people residing on Indian reserves with Aboriginal people residing off-reserve, or with the general population. We classified primary care visits by injury type (trauma, poisoning, burn, or other), according to the diagnostic code. Unlike death certificates and hospital discharge records, each MSP payment record contains only a single diagnostic code, therefore it cannot also contain a supplemental ICD-9 “E” code describing the external cause of the injury. Also, as of December 31, 2010, MSP payment records continue to use the ICD-9 coding system. We tabulated numbers of visits by injury type, calendar year (of visit date), gender, 5-year age group, Aboriginal status, reserve residence, HSDA, and metropolitan residence.

### Incidence rates of primary care visits

We calculated the *crude rate* of primary care visit as the number of visits divided by the person-years of observation (the sum of the annual population counts) during the same time period. We considered the crude rate to be a binomial proportion, and we estimated standard errors of the proportion, and 95 % confidence intervals of the proportion accordingly. We calculated *Standardized Relative Risk* (SRR) of primary care visit relative to the risk in the reference population (82,585,786 person-years, the combined total population of BC from January 1, 1991 through December 31, 2010) using the method of indirect standardization [11, 26], standardizing by gender, 5-year age group, and HSDA, or standardizing for just gender and 5-year age group when calculating SRRs for specific HSDAs, or for HSDAs aggregated into categories (metropolitan or not metropolitan).

We assessed cumulative change in SRR over time as the proportional change between the first and last years of the observation period, i.e., (SRR_2010_/SRR_1991_) −1. To facilitate comparisons, we converted proportional change over the entire period to an annualized change, using this formula.

$$ {\left(\frac{SR{R}_{2010}}{SR{R}_{1991}}\right)}^{1/\left(2010-1991\right)}-1 $$

## Results

### Aboriginal status and off-reserve residence

Table 1 shows crude rates and SRRs of primary care visits due to injury, during the period 1991–2010, among the total population of BC (i.e., the reference population), and the Aboriginal population residing off-reserve. For every major category of injury type, the crude incidence and the SRR (standardized by age, gender and HSDA) are higher among the Aboriginal off-reserve population than among the total population.

**Table 1:** Primary care visits^a^ due to injuries^b^, British Columbia, 1991-2010^c^

	Obs^d^	Rate^e^	95 % CI for Rate	SRR^f^	95 % CI for SRR
BC, total population					
Total, All injuries	26,194,409	3172	3171 to3173	1	[reference]
Trauma	22,873,669	2770	2769 to2771	1	[reference]
Poisoning	712,214	86	86 to 86	1	[reference]
Burn	496,325	60	60 to 60	1	[reference]
Other	2,112,201	256	255 to 256	1	[reference]
Aboriginal, off-reserve^g^					
Total, All injuries	640,458	4291	4283 to 4299	1.41	1.41 to 1.42
Trauma	545,482	3655	3647 to 3663	1.40	1.40 to 1.40
Poisoning	31,044	208	206 to 210	2.08	2.05 to 2.12
Burn	14,221	95	94 to 97	1.28	1.25 to 1.30
Other	49,711	333	330 to 336	1.31	1.30 to 1.33

^a^ “Primary care” defined as examination by general practice physician, emergency physician, nurse practitioner, pediatrician, geriatrician, dentist, or optometrist

^b^ “Injury” defined as Diagnosis in the range ICD9:800–999

^c^Medical Service Plan payments occurring during the observation period 1991-Jan-01 to 2010-Dec-31

^d^Observed number of MSP payments

^e^Crude Rate per 10,000 person-years = Observed / Person-years x 10,000, (person-years is the sum of annual population counts during the observation period)

^f^Standardized Relative Risk (compared to the total population of BC during the same observation period) = Observed/Expected, (expected number is standardized by age, gender and HSDA)

^g^Aboriginal identity deduced from birth record, death record or MSP premium payment record; and residing in a postal code area that does not contain any Indian reserve

### HSDAs and metropolitan residence

Table 2 shows crude rates and age and gender-standardized SRRs of primary care visit due to injury, during the period 1991–2010, within the total population and the Aboriginal off-reserve population of each HSDA. Crude incidence rates and SRRs are highly variable among HSDAs, and this applies to both population groups. Comparing crude incidence rates within specific HSDAs, one sees that in most, but not all HSDAs, the Aboriginal off-reserve population has a higher rate of primary care visit due to injury than do the total population. Comparing SRRs, one sees that in every HSDA, standardized for age and gender, Aboriginal off-reserve people have a higher risk of primary care visit due to injury than do the total population.

**Table 2:** Primary care visits^a^ due to injuries^b^, British Columbia, 1991-2010^c^, by Health Service Delivery Area

	Total population					Aboriginal off-reserve population				
HSDA	Obs^d^	Rate^e^	95 % CI for Rate	SRR^f^	95 % CI for SRR	Obs^d^	Rate^e^	95 % CI for Rate	SRR^f^	95 % CI for SRR
11	444,368	2825	2818 to 2832	0.90	0.90 to 0.91	6685	3292	3228 to 3357	1.14	1.12 to 1.17
12	550,752	3445	3437 to 3452	1.09	1.09 to 1.09	4725	3855	3769 to 3942	1.33	1.30 to 1.37
13	2,086,611	3340	3337 to 3344	1.05	1.05 to 1.05	31,048	3936	3902 to 3970	1.35	1.34 to 1.37
14	1,463,207	3421	3416 to 3425	1.10	1.10 to 1.10	61,471	4399	4373 to 4425	1.50	1.49 to 1.51
21	1,680,239	3491	3487 to 3496	1.12	1.12 to 1.12	42,505	4580	4548 to 4612	1.60	1.58 to 1.61
22	3,164,447	3008	3005 to 3011	0.96	0.96 to 0.96	33,551	3477	3447 to 3507	1.20	1.19 to 1.22
23	4,673,288	3956	3953 to 3959	1.27	1.27 to 1.28	40,444	3833	3804 to 3862	1.37	1.36 to 1.38
31	870,825	2483	2478 to 2488	0.79	0.79 to 0.79	5400	3628	3551 to 3705	1.25	1.22 to 1.28
32	3,231,405	2704	2701 to 2706	0.85	0.85 to 0.85	81,118	3782	3762 to 3803	1.26	1.26 to 1.27
33	1,457,661	2770	2766 to 2774	0.88	0.88 to 0.88	17,722	4004	3958 to 4049	1.36	1.34 to 1.38
41	1,731,570	2567	2564 to 2570	0.80	0.80 to 0.80	32,297	3453	3422 to 3483	1.18	1.16 to 1.19
42	1,570,298	3268	3264 to 3272	1.04	1.03 to 1.04	69,506	4504	4479 to 4529	1.55	1.54 to 1.57
43	910,131	3961	3955 to 3968	1.27	1.27 to 1.28	30,614	5568	5527 to 5610	1.92	1.89 to 1.94
51	627,990	3700	3692 to 3707	1.21	1.21 to 1.22	90,795	5253	5229 to 5277	1.78	1.77 to 1.79
52	1,169,896	3885	3880 to 3891	1.27	1.27 to 1.27	63,010	4895	4867 to 4922	1.68	1.67 to 1.70
53	380,109	2878	2870 to 2886	0.95	0.95 to 0.95	26,124	4426	4386 to 4466	1.53	1.51 to 1.55
Metro^g^	15,129,196	3038	3037 to 3039	0.96	0.96 to 0.97	210,532	3699	3686 to 3711	1.27	1.26 to 1.27
Not^h^	10,883,601	3440	3438 to 3441	1.10	1.10 to 1.10	426,483	4667	4657 to 4677	1.60	1.60 to 1.61
All HSDAs	26,012,797	3194	3193 to 3195	1	[reference]	637,015	4295	4287 to 4303	1.47	1.47 to 1.48

^a^ “Primary care” defined as examination by general practice physician, emergency physician, nurse practitioner, pediatrician, geriatrician, dentiist, or optometrist

^b^ “Injury” defined as Diagnosis in the range ICD9:800–999

^c^Medical Service Plan payments occurring during the observation period 1991-Jan-01 to 2010-Dec-31

^d^Observed number of MSP payments

^e^Crude Rate per 10,000 person-years

^f^Standardized Relative Risk (compared to the total population of BC during the same observation period) = Observed/Expected

^g^Metropolitan Vancouver and Victoria: aggregation of HSDAs 22, 23, 31, 32, 33 and 41

^h^Not metropolitan: aggregation of HSDAs 11, 12, 13, 14, 21, 42, 43, 51, 52, and 53

Aggregating the HSDAs into categories of “metropolitan” or “not metropolitan”, one sees that metropolitan HSDAs have lower crude rates and SRRs than do HSDAs that are not metropolitan, and this applies to both the Aboriginal off-reserve population and among the total population. Metropolitan-dwelling Aboriginal off-reserve people have a higher risk of primary care visit due to injury than do the metropolitan dwelling total population. Aboriginal off-reserve people residing in non-metropolitan HSDAS also have a higher risk of primary care visit due to injury than do the total population residing in non-metropolitan HSDAs.

Aggregating all HSDAs together, the Aboriginal off-reserve population of BC has SRR of 1.47, standardized for age and gender. Compare this to the SRR of 1.41 (standardized for age, gender and HSDA) for the same population, with all injury categories combined, shown in Table 1.

### Age and gender

Table 3 shows crude rates and age and gender-specific, HSDA-standardized SRRs of primary care visit due to injury, during the period 1991–2010, within specific age and gender categories of the total population and the Aboriginal off-reserve population of BC. Among those aged less than 50 years, males have higher incidence rates of primary care visit due to injury than do females, but among those aged 50 years and older, females have higher incidence rates, and the excess among females is even larger among persons aged 70 years or older. This pattern applies both among the total population and among the Aboriginal off-reserve population. Among females, the incidence rate increases with age, with this pattern occurring in both the total population and the Aboriginal off-reserve population. Among the males in the Aboriginal off-reserve population, the incidence rate increases with age, peaking in the 40 to 49 year age group, then declines. Among males in the total population, the rate peaks in the 10 to 19 year age group, declines, then increases again in males over 70 years of age.

**Table 3:** Primary care visits^a^ due to injuries^b^, British Columbia, 1991-2010^c^, by gender and age

		Total population				Aboriginal off-reserve population				
Gender	Age	Obs^d^	Rate^e^	95 % CI for Rate	SRR [ref]	Obs^d^	Rate^e^	95 % CI for Rate	SRR^f^	95 % CI for SRR
F	0–9	887,641	1847	1843 to 1850	1	38,863	2155	2136 to 2174	1.13	1.13 to 1.13
F	10–19	1,467,795	2835	2831 to 2839	1	43,526	3436	3410 to 3463	1.17	1.17 to 1.17
F	20–29	1,558,581	2754	2751 to 2758	1	54,821	4318	4291 to 4345	1.52	1.52 to 1.52
F	30–39	1,888,758	2930	2926 to 2933	1	66,278	5079	5052 to 5106	1.68	1.68 to 1.68
F	40–49	2,024,234	3095	3092 to 3099	1	56,868	5557	5526 to 5587	1.77	1.77 to 1.78
F	50–59	1,648,124	3298	3294 to 3302	1	33,206	5544	5504 to 5584	1.66	1.65 to 1.66
F	60–69	1,102,710	3133	3128 to 3138	1	15,822	5188	5132 to 5244	1.59	1.58 to 1.60
F	70–79	1,038,629	3899	3893 to 3905	1	8040	5905	5822 to 5988	1.44	1.42 to 1.46
F	80+	1,187,321	6418	6412 to 6425	1	4032	6118	6000 to 6235	0.91	0.89 to 0.93
M	0–9	1,112,452	2199	2196 to 2203	1	47,416	2517	2497 to 2536	1.10	1.10 to 1.10
M	10–19	2,062,643	3772	3768 to 3776	1	53,712	4148	4121 to 4175	1.06	1.06 to 1.06
M	20–29	2,028,255	3624	3621 to 3628	1	58,897	5193	5164 to 5223	1.37	1.37 to 1.37
M	30–39	2,225,909	3516	3512 to 3520	1	66,317	5734	5705 to 5762	1.58	1.58 to 1.58
M	40–49	2,174,197	3337	3334 to 3341	1	51,154	5977	5944 to 6010	1.74	1.74 to 1.75
M	50–59	1,583,621	3142	3138 to 3146	1	24,185	5374	5328 to 5420	1.66	1.65 to 1.66
M	60–69	985,674	2836	2831 to 2840	1	10,905	4997	4931 to 5064	1.68	1.67 to 1.70
M	70–79	694,262	3053	3047 to 3059	1	4248	4587	4486 to 4689	1.44	1.39 to 1.48
M	80+	499,542	4549	4540 to 4558	1	1955	4644	4493 to 4795	0.99	0.92 to 1.08

^a^ “Primary care” defined as examination by general practice physician, emergency physician, nurse practitioner, pediatrician, geriatrician, dentist, or optometrist

^b^ “Injury” defined as Diagnosis in the range ICD9:800–999

^c^Medical Service Plan payments occurring during the observation period 1991-Jan-01 to 2010-Dec-31

^d^Observed number of MSP payments

^e^Crude Rate per 10,000 person-years

^f^Standardized Relative Risk (compared to the total population of BC during the same observation period) = Observed/Expected

Comparing age and gender-specific crude incidence rates, one sees that all age and gender categories except females aged 80 years or older), Aboriginal off-reserve people have a higher rate of primary care visit due to injury than do the total population. Comparing age and gender-specific, HSDA-standardized SRRs, one sees that in every age and gender category except the most elderly (females and males aged 80 years or older) Aboriginal off-reserve people have a higher risk of primary care visit due to injury than do the total population. The increased relative risk among Aboriginal off-reserve people is less severe among the young (less than 20 years of age).

### Changes over time

**Fig. 1:**
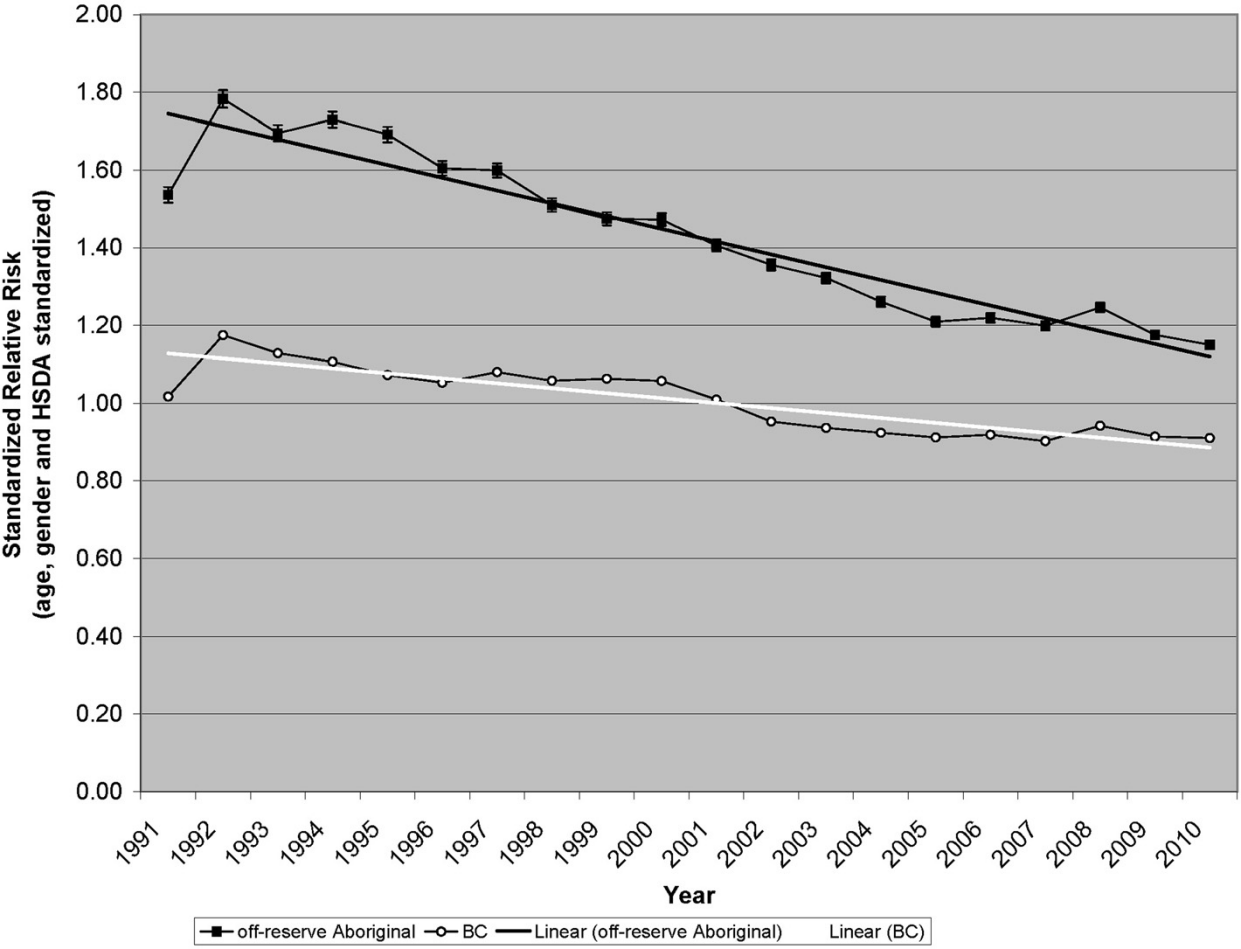
Primary care visits due to injuries, British Columbia, 1991–2010, by calendar year

Figure 1 shows SRRs of primary care visit due to injury, during the period 1991–2010, among the total population and the Aboriginal off-reserve population of BC, by year. SRRs have been standardized for age, gender, and HSDA. Recall that the reference population is the combined total population of BC during the entire period (1991 through 2010). Thus, the SRR for the total population in a particular year can be higher or lower than one, but the average of the SRRs for the total population, over all the years, will be equal to one. The Aboriginal off-reserve population had higher SRR than did the total population, in all years. Over the period 1991–2010, there was substantial decrease of SRR among both populations.

According to our analysis of the population registry, among the Aboriginal off-reserve population, the proportion residing in metropolitan HSDAs decreased from 39.8 % in 1991 to 36.3 % in 2010. In contrast, among the total population of BC, the proportion increased from 60.0 % in 1991 to 62.4 % in 2010. Having standardized by HSDA, we can say that the reductions in SRR are independent of any effect from changes in the proportions of the populations residing in metropolitan areas.

Table 4 shows proportional changes in SRR between 1991 and 2010, among the Aboriginal off-reserve and the total population of BC, by categories of injury type. Between 1991 and 2010 there was 25.2 % decline in SRR of primary care visit due to injury (total, all types combined) among the Aboriginal off-reserve population (annualized change of −1.5 %), compared to 10.5 % decline among the total population (annualized change of −0.6 %). The disparity between the Aboriginal off-reserve and total populations was statistically significant (p < 0.001, 2-sided). In all major categories of injury type (trauma, poisoning, and burn) the Aboriginal off-reserve population showed significantly larger proportional declines in SRR than did the total population.

**Table 4:** Standardized Relative Risks of primary care visit due to injury, British Columbia, 1991–2010

Population; Injury type	SRR^a^ 1991	SRR^a^ 2010	1991 to 2010 % change	p^b^	Annual % change	L95CL	U95CL
Aboriginal, off-reserve							
Total, All injuries	1.54	1.15	−25.2 %	0.000	−1.5 %	−1.6 %	−1.4 %
Trauma	1.48	1.14	−23.1 %	0.000	−1.4 %	−1.5 %	−1.3 %
Poisoning	2.88	1.84	−36.0 %	0.044	−2.3 %	−2.9 %	−1.8 %
Burn	1.94	0.77	−60.3 %	0.000	−4.8 %	−5.4 %	−4.1 %
Other injury type	1.49	1.12	−24.8 %	0.004	−1.5 %	−1.8 %	−1.1 %
BC							
Total, All injuries	1.02	0.91	−10.5 %	NA	−0.6 %	−0.6 %	−0.6 %
Trauma	0.99	0.92	−6.4 %	NA	−0.3 %	−0.4 %	−0.3 %
Poisoning	1.26	0.90	−28.7 %	NA	−1.8 %	−1.8 %	−1.7 %
Burn	1.22	0.68	−44.6 %	NA	−3.1 %	−3.1 %	−3.0 %
Other injury type	1.23	0.84	−31.8 %	NA	−2.0 %	−2.0 %	−1.9 %

^a^SRR standardized by age, gender and HSDA, relative to total population of BC, 1991 to 2010

^b^probability (2-sided, z-test) that Ln((SRR 2010)/(SRR 1991)) Aboriginal = Ln((SRR 2010)/(SRR 1991)) BC total

As shown in Table 5, metropolitan residents showed larger declines in SRR of trauma, poisoning, burn than did non-metropolitan residents. This pattern occurred in both the Aboriginal off-reserve and the general population. The greater declines in SRR means that the gap between the Aboriginal off-reserve and total populations is shrinking, particularly among metropolitan residents.

**Table 5:** Standardized Relative Risks of primary care visit due to injury (categorized by type and metropolitan residence), British Columbia, 1991–2010

Population; Injury type	SRR^a^ 1991	SRR^a^ 2010	1991 to 2010 % change	p^b^	Annual % change	L95CL	U95CL
Aboriginal off-reserve, not metropolitan							
Total, All injuries	1.58	1.35	−14.4 %	0.000	−0.8 %	−0.9 %	−0.7 %
Trauma	1.51	1.33	−12.1 %	0.000	−0.7 %	−0.8 %	−0.6 %
Poisoning	2.57	2.13	−17.4 %	0.240	−1.0 %	−1.7 %	−0.3 %
Burn	2.30	0.99	−57.1 %	0.000	−4.4 %	−5.2 %	−3.5 %
Other injury type	1.72	1.43	−17.3 %	0.096	−1.0 %	−1.5 %	−0.5 %
Aboriginal off-reserve, metropolitan							
Total, All injuries	1.59	0.96	−39.4 %	0.000	−2.6 %	−2.7 %	−2.5 %
Trauma	1.52	0.95	−37.7 %	0.000	−2.5 %	−2.6 %	−2.3 %
Poisoning	3.70	1.73	−53.3 %	0.007	−3.9 %	−4.8 %	−3.0 %
Burn	1.79	0.62	−65.3 %	0.000	−5.4 %	−6.4 %	−4.4 %
Other injury type	1.42	0.91	−36.0 %	0.902	−2.3 %	−2.9 %	−1.7 %
BC, not metropolitan							
Total, All injuries	1.01	1.03	2.2 %	NA	0.1 %	0.1 %	0.1 %
Trauma	0.95	1.03	7.9 %	NA	0.4 %	0.4 %	0.4 %
Poisoning	1.32	1.18	−10.2 %	NA	−0.6 %	−0.7 %	−0.4 %
Burn	1.43	0.84	−41.4 %	NA	−2.8 %	−2.9 %	−2.6 %
Other injury type	1.44	1.10	−23.3 %	NA	−1.4 %	−1.5 %	−1.3 %
BC, metropolitan							
Total, All injuries	1.02	0.85	−16.4 %	NA	−0.9 %	−0.9 %	−0.9 %
Trauma	1.00	0.87	−12.7 %	NA	−0.7 %	−0.7 %	−0.7 %
Poisoning	1.23	0.73	−40.5 %	NA	−2.7 %	−2.8 %	−2.6 %
Burn	1.10	0.59	−46.5 %	NA	−3.2 %	−3.4 %	−3.1 %
Other injury type	1.08	0.69	−36.4 %	NA	−2.4 %	−2.4 %	−2.3 %

^a^SRR standardized by age and gender, relative to total population of BC, 1991 to 2010

^b^probability (2-sided, z-test) that Ln((SRR 2010)/(SRR 1991)) Aboriginal = Ln((SRR 2010)/(SRR 1991)) BC

## Discussion

Aboriginal off-reserve people have higher incidence of primary care visit due to injury than the total population of BC. Standardizing for age reveals the disparity that was masked because the Aboriginal off-reserve population are on average younger than the total population, and primary care visit due to injury is most frequent among the elderly. Standardizing for geographic area of residence (HSDA) eliminates the confounding that tended to exaggerate the disparity, because the Aboriginal off-reserve population are more likely to reside in northern or non-metropolitan HSDAs, where primary care visit due to injury occurs more frequently. Standardizing for both age and HSDA takes into account these competing effects, and we found that Aboriginal off-reserve people have 1.41 times the risk of primary care visit due to injury than the total population. Changes over time in risk of primary care visit due to injury suggest that the gap between the Aboriginal off-reserve population and the total population is narrowing, and more rapidly among metropolitan residents.

Standardizing by age and gender (but not HSDA), Aboriginal off-reserve people have 1.47 times the risk of primary care visit due to injury than the total population. Therefore, we estimate that 13 % [i.e., (1.47–1.41)/(1.47–1)] of the age and gender-standardized risk disparity between the Aboriginal off-reserve and total populations of BC is attributable to geography, and the remaining 87 % is attributable to other factors. In a future series of reports, we will measure the extent to which socioeconomic conditions, as well geography, can explain the disparities of injury risk between the Aboriginal and total populations of BC.

The patterns of disparity in risk of primary care visit due to injury are remarkably similar to those we reported previously, regarding risk of *hospitalization* due to injury. The changes over time are similar [10], as are the patterns by gender, age group and metropolitan residence regarding risk of hospitalization due to unintentional falls [11], comparing the Aboriginal population to the total population of BC. This suggests that our observations, now and in previous reports [8–11], are not merely artefacts related to health care utilization, but reflect real disparities and trends in the underlying incidence of injury.

In both the present analysis of primary care visits due to injury and our previous analysis of hospitalizations due to unintentional falls [11], we observed that the increased relative risk among Aboriginal off-reserve people (compared to the general population) is less severe among the young (less than 20 years of age) and the very elderly. This suggests that the socioeconomic and environmental disadvantages faced by Aboriginal people may have been partially mitigated by prevention programs and cultural adaptations aimed at protecting children. The apparently reduced risk among the very elderly may simply indicate a survivorship bias, due to earlier attrition of susceptible individuals within the Aboriginal population. However, the secular trend towards declining injury rates [10], likely driven by improvements in socioeconomic conditions [27], favours older adults more than the young [11].

The disparities in risk of primary care visit due to injury among the Aboriginal off-reserve population, compared to the general population, though statistically and materially significant, were much smaller than the disparities in risk of death [18], and somewhat smaller than the disparities in risk of hospitalization [11]. Aboriginal people may have more severe injuries than the general population, leading to death without hospitalization, or hospitalization instead of primary care treatment. Alternatively, Aboriginal people may under-utilize health care services for less severe injuries.

Primary care visits are an indicator of injury burden, but are also influenced by availability and access to primary health care. These factors vary among regions of the province, and we standardized risks by HSDA, so the comparison between the Aboriginal off-reserve and the total populations of BC should not be biased. As we explained above, we excluded residents of Aboriginal reserves from this analysis, because the usual method of payment for on-reserve health care services would have introduced a measurement bias that we could not have compensated for.

The main limitations of this study are in the outcome that we measured. In this study we did not count injuries, we counted primary care visits due to injury. An injury often entails more than one primary care visit. Primary care visits would include minor injuries requiring no further care, but also severe injuries requiring hospitalization, surgery or other specialist care, as a primary care practitioner may have performed the initial assessment, and likely would also be involved with subsequent care. Generally, more severe injuries would be associated with greater numbers of visits, but extremely severe injuries resulting in immediate death would not require any primary care visit. Primary care visits, hospitalizations [10, 11], worker compensation claims [9], and deaths [2, 4, 18] are all pieces of a larger picture.

Another limitation is that the Aboriginal population we studied included only off-reserve residents. In future reports, measuring the extent to which socioeconomic markers, geographic place, and Aboriginal ethnicity explain disparities of injury risk, we will count injury hospitalizations as the outcome of interest, and we will include both on-reserve and off-reserve Aboriginal people.

## Conclusions

The historical and persistent disparity in socioeconomic status, and health and safety outcomes between the Aboriginal and general populations is a significant moral and public policy challenge for Canada. Measuring health and safety in the starkest of terms, death, the disparity is most egregious in the category of injury [2]. The causes and possible remedies to the disparity are important research questions. Our research on injuries contributes to a broader discussion.

## Appendix

**Table 6:** Injury categories derived from International Classification of Diseases and Medical Services Plan of BC codes

Injury category	ICD-9 codes	MSP codes
All injury types	800–999	02B (skin graft)
		10A (assault, emergency care)
		22B (open wound female genital)
		42A (cast removal)
		43A (change dressing)
		55B (foreign body hand/finger)
		60B (foreign body foot/toe)
		65B (animal bite)
		66B (insect bite)
Trauma	800–908, 910–939, 950–959	10A, 22B, 42A, 55B, 60B, 65B
Poisoning	909.0, 909.1, 960–989	66B
Burn	940–949	
Other injury types	909.2–909.9, 990–999.9	02B, 43A
